# Risk Assessment of the Progression of Early Knee Osteoarthritis by Collagen Neoepitope C2C: A Longitudinal Study of an Estonian Middle-Aged Cohort

**DOI:** 10.3390/diagnostics11071236

**Published:** 2021-07-10

**Authors:** Liisa Kuhi, Ann E. Tamm, Agu O. Tamm, Kalle Kisand

**Affiliations:** 1 Department of Internal Medicine, Institute of Clinical Medicine, University of Tartu, 50090 Tartu, Estonia; agu.tamm@kliinikum.ee (A.O.T.); kalle.kisand@ut.ee (K.K.); 2 Central Laboratory, Diagnostic Clinic, East-Tallinn Central Hospital, 10138 Tallinn, Estonia; 3 Sports Medicine and Rehabilitation Clinic, Institute of Clinical Medicine, University of Tartu, 50090 Tartu, Estonia; ann.tamm@kliinikum.ee

**Keywords:** osteoarthritis, knee, osteophytes, joint space narrowing, neoepitope C2C, biomarker, prognostic

## Abstract

One of the unmet needs to be addressed is prognostic biomarkers for early knee osteoarthritis (kOA). We aimed to study the association of urinary collagen type-II C-terminal cleavage neoepitope (uC2C) with the emergence and progression of kOA. The longitudinal data of 330 subjects (aged 32–60 years) from an Estonian population-based cohort were used. The radiographic progression was evaluated by the grading system of Nagaosa et al. of knee compartments at baseline and three years later. The emerging kOA consisted of subjects with developing osteophytes or joint space narrowing, whereas kOA progressors showed aggravation of radiographic grade. Baseline uC2C levels were measured by the IBEX-uC2C assay. At baseline, the subjects were middle-aged (mean age, 47.6 years) and overweight (mean BMI, 28.0 kg/m^2^), and the majority of them (51.2%) had a diagnosis of kOA grade 1. Multiple logistic regression models adjusted for sex, age, and BMI were used for risk calculations. We demonstrate that increased uC2C accurately predicted the risk of emerging of kOA (OR = 5.87 (1.71–20.22); AUC = 0.79) compared with controls without radiographic kOA over 12 years. However, the most accurate prediction of progression by the biomarker was found in women (OR = 23.0 (2.2–245), AUC = 0.91). In conclusion, uC2C may be a promising candidate as a prognostic biomarker for kOA progression, particularly of emerging kOA in women.

## 1. Introduction

Osteoarthritis (OA) is considered a whole joint disease that involves the articular cartilage, synovial tissues, subchondral bone, ligaments, and meniscus, leading to a reduction in quality of life and disability [1,2]. Knee OA (kOA) is highly prevalent today because of numerous factors, including aging and obesity, and the prevalence is expected to increase [3,4]. To date, the complexity of OA pathogenesis has become apparent. It is unclear from which joint tissue (synovia, meniscus, subchondral bone, or cartilage) or which part of the joint, TF or PF compartment, the disease begins or which are the drivers of disease progression [5,6]. It was proposed that different phenotypes of kOA rather than disease status are responsible for the type of disease progression [7]. Therefore, it was suggested that a combination of different biomarkers that can reflect distinct aspects of metabolism of knee tissues should be used in both diagnosis and prognosis of OA [8].

A critical point for managing the increasing burden of this chronic, disabling disorder is to identify the subjects at high risk of kOA progression. In addition to imaging-based markers, several studies for discovering the soluble prognostic markers of bone and cartilage turnover have been conducted [9,10,11]. The Osteoarthritis Research Society International-FDA (OARSI-FDA) Biomarkers Group identified nine biomarkers that were able to predict case status, reflecting clinically relevant progression over 48 months, among which was human urinary collagen type II (Col2) C-terminal cleavage neoepitope (uC2C) [8]. The potential of this Col2 degradation marker for the prediction of knee OA worsening was also described independently in two other studies [12,13]. The uC2C (IB-C2C-HUSA, Ibex) ELISA assay was developed to specifically detect the OA pathology-related 45 mer peptide, the C2C neoepitope of type II collagen in urine. This sandwich assay is more predictive of the progression of cartilage degeneration compared with the previous C2C competitive inhibition assays [13]. However, a previous study commented that the assays are able to detect the pathology-related collagen neopeptide in the urine, but little or no reactivity was seen in the serum. Therefore, uC2C evaluation requires normalization to urine concentration using urine creatine values [12].

Although the previous studies described the association between uC2C and radiographic progression of kOA, to the best of our knowledge, no studies have examined the association of the biomarker through the course of the disease or, in particular, the ability to predict the early progressors and the emergence of kOA. The prediction of an early disease progression is particularly important not only to manage patients but also to develop new drugs and early treatment interventions [14,15]. 

In our previous study, we demonstrated a significant difference in uC2C levels between sex and kOA grades [16], but the influence of this factor on the prediction is unknown. Thus, we aimed in this study to evaluate whether uC2C values: (1) can be an early prognostic risk marker, (2) are associated with progression in distinct kOA severity grade, (3) can be the prognostic marker for cases with minimal kOA progression, and (4) show any sex differences between uC2C prognostic values.

## 2. Materials and Methods

### 2.1. Subjects

In this longitudinal study, a total of 330 subjects aged 32–60 years, recruited by general practitioners or visited orthopedic surgeons (described previously in detail [17,18]), were investigated. The follow-up period for the study group was about three years (mean of 38 ± 5 months). Demographic, clinical data, and radiographs were obtained for all subjects at two time-points: baseline (T0) and follow-up (T3) three years later. The subjects under 65 years of age with the ability to attend all ambulatory visits were included. The knee injury or arthroscopic surgery were not exclusion criteria. The urine samples were collected and assessed at T0. Subjects’ pain in joints was evaluated using a visual analogue scale (VAS). Two mean VAS scores were calculated: (a) for knee joints (mean knee VAS score); (b) for other joints (hands (finger/palm+shoulder+elbow), hips, ankle+foot, spine (neck and lower back), jaw joint, the median summary VAS score). Subjects’ knee symptoms were evaluated using the Knee Injury and Osteoarthritis Outcome Score (KOOS) [19]. Subjects with radiographic evidence of rheumatoid arthritis or other inflammatory arthropathies in the knees, history of knee arthroplasty, or technically unsuitable radiographs were excluded. The subjects without knee complaints (pain and/or stiffness), KOOS ≥85% at baseline, and no radiographic kOA changes during the follow-up period were considered to have healthy knees.

The Research Ethics Committee of the University of Tartu approved the study, which was conducted according to the precepts of the Declaration of Helsinki. The subjects provided written informed consent for study participation.

### 2.2. Radiographic Evaluation

Standardized anteroposterior radiographs of tibiofemoral (TF) joints and radiographs of patellofemoral (PF) joints with the knees at 60° flexion were evaluated for all study subjects. Details of the radiographic evaluation were published elsewhere [20]. Briefly, two radiologists independently graded joint space narrowing (JSN) and osteophytes (Oph) formation in the two joints, the tibiofemoral (TF) and patellofemoral (PF), using the Nottingham system (grades 0–3) [21]. For bilateral cases, the knee with more severe OA served as the study knee. The highest grade of OA changes among all of each subject’s knee compartments is expressed as the radiographic global grade of knee osteoarthritis (gOA). 

### 2.3. Definitions of Progression and Distribution of the Radiographic Groups 

The progression of kOA was evaluated by the comparison of their radiographic findings at time points T0 and T3. Two main outcome groups were defined: (1) the progressors group, which was formed by the subjects with signs of radiographic OA progression in knee joint during three years; and (2) the non-progressors group, which consisted of the subjects lacking the radiographic progression of knee OA. The progressors group was divided into two subgroups according to the extent of the changes: (a) gr(ade)-progressors, which included the subjects with radiographic worsening of at least one grade of gOA (≥1 grade) within 3 years; (b) the min(imal)-progressors, which contained the subjects with radiographic worsening within the same gOA grade (addition or increasing the grade of Oph or JSN in the TF or PF joint within 3 years). The subjects in gOA grade 0 at baseline but developing kOA grade 1 or more during the three-year period, regarding the incidence of kOA, were named the emerging kOA group. The subjects without radiographic kOA during the whole study period (grade 0 of gOA) were defined as the without-kOA group. The subjects without knee complaints, KOOS ≥85% at baseline, and no radiographic kOA changes during the twelve-year follow-up period formed a long-term control group.

### 2.4. uC2C Measurement 

The study subjects were instructed to collect urine from the second morning void. Urine samples were stored at −80 °C on the day of collection. The concentrations of C2C neoepitope fragments were measured in urine using the IBEX C2C human urine sandwich assay (IB-C2C-HUSA) by IBEX Pharmaceuticals (IBEX Pharmaceuticals Inc., Montreal, Quebec, Canada). The assay details and performance characteristics were described by Poole et al. [13] and https://www.ibex.ca/product-catalog/, 15 May 2018. All samples were tested in duplicate, and each measured C2C concentration was corrected with the creatinine concentration in the same urine sample, determined using the QuantiChrom™ Creatinine Assay kit (DICT-500; BioAssay Systems, Hayward, USA).

### 2.5. Statistical Analysis

The sample size was calculated using a sample size calculator for Mann–Whitney test with confidence interval set at 95% and power at 80%, and the results obtained were 100 in progressors and non-progressors for the whole study group.

The data were analyzed using R (version 3.4.3; Free Software Foundation, Boston, MA, USA; http://www.r-project.org, 7 December 2018). Descriptive variables at baseline are presented as a number or mean with standard deviation and were compared using analysis of variance. The percentages of subjects in different gOA grade groups were compared using the chi-square test. As the uC2C concentration data were not distributed normally, medians and 25th and 75th percentiles for each radiographic progression group were calculated. The uC2C data were analyzed using the Kruskal–Wallis rank-sum test and the Mann–Whitney U-test. *p*-values < 0.05 were considered significant.

The multiple logistic regression with the forward selection method for independent variables was used to compare the following groups: (1) progressors vs. non-progressors in all grades; (2) gr-progressors vs. non-progressors in all grades; (3) emerging kOA (baseline grade 0) vs. without-kOA; (4) emerging kOA in grade 0 vs. the long-term control group; (5) progressors vs. non-progressors in grade 1; and (6) progressors vs. non-progressors in grades 2 or 3. These comparisons were performed for the whole group and the sexes separately. As several confounders may influence the course of kOA, each model was adjusted for age, sex, and body mass index (BMI). The models containing the subjects of all grades were adjusted for gOA because the level of uC2C is associated with gOA severity grade [22]. The discriminative ability of uC2C was assessed using the c statistic (area under the curve (AUC)), and receiver-operating characteristic (ROC) curves were generated for each of the comparisons.

## 3. Results

### 3.1. Distribution of the Radiographic Groups

**Figure 1:**
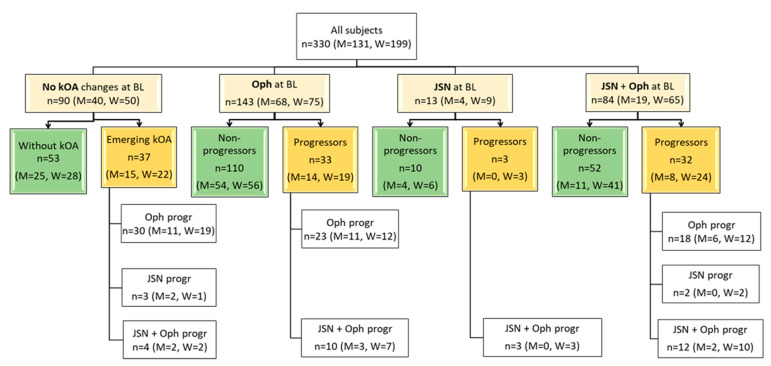
Baseline/initial (T0) radiographic status of the knees of the cohort and type of progression 3 years later (T1). BL, baseline; Oph, osteophytes; JSN, joint space narrowing; Oph + JSN, osteophytes and joint space narrowing at the same time; Progressors—subjects with the progression of knee osteoarthritis (kOA) during 3 years; Non-progressors—subjects without radiographical changes of kOA during 3 years; Oph progr—radiographic progression of osteophytes; JSN progr—radiographic progression of joint space narrowing; Oph+JSN progr—radiographic progression of osteophytes and joint space narrowing; n, numbers, M, numbers in men, W, numbers in women.

According to the definitions, the distribution of the subjects by radiographic groups is shown in Table 1 and by radiographic signs in Figure 1. 

The most prevalent group at baseline consisted of the subjects with early grade (1) of kOA (51.2% of all cases). Moreover, osteophytosis was the most frequent (43.3%) radiographic sign of kOA in the cohort and the most frequent sign that defined the progression of the disease (in 67.6% of progressors). The next most-frequent marker of progression was the combined aggravation of Oph and JSN (27.6% of progressors). More than half (60.3%) of the subjects were women, and the distribution of radiographic findings among sexes at baseline as well as in progression were similar. The long-term control group contained 24 of the subjects. Notably, a small under-representation of women was observed in the long-term control group (42% women).

### 3.2. Baseline Characteristics of the Study Group

At T0, the subjects of the study group were middle-aged (aged 32–56 years, mean age, 47.6 ± 6.5 years) and overweight (mean BMI, 28.0 ± 5.3 kg/m^2^). The clinical characteristics of the subjects by progression status, gOA grades, and sex are presented in Table 2. 

The mean age as well as BMI had the highest values in gOA grade 2. The mean age of men was slightly lower (1.8 years) compared with that of women (*p* = 0.04), and the mean age of male progressors was 3.1 years lower compared with female progressors (46.0 ± 6.3 years vs. 49.1 ± 6.0 years, respectively, *p* = 0.02). We observed higher BMI in progressors at T0 compared with non-progressors in the whole group (*p* = 0.003) as well as in women (*p* = 0.008). In contrast, the BMI in men showed no differences between progression subgroups. Interestingly, men without kOA were slightly overweight compared with women without kOA (27.7 ± 5.4 kg/m^2^ vs. 24.1 ± 3.9 kg/m^2^, respectively, *p* = 0.007).

The mean age of the subjects in the long-term control group was 49.1 ± 5.7 years (48.9 ± 6.3 years in men and 49.4 ± 5.1 years in women), and the BMI was 26.0 ± 4.6 kg/m^2^. The men’s BMI was 15% higher compared with that of women (27.7 ± 5.0 kg/m^2^ and 23.6 ± 2.4 kg/m^2^, respectively, *p* = 0.01). Thus, the characteristics of the control group and the total gOA grade of the 0 group did not differ significantly. 

The median summary VAS score of other joints was 0.3 (CI 95% 0–0.8). The mean value of C-reactive protein using the high-sensitive method was 2.2 ± 2.5 mg/L. The median summary VAS score and the mean value of C-reactive protein did not differ between progressors and non-progressors (*p* = 0.3 and *p* = 0.8, respectively).

### 3.3. Association of uC2C Baseline Level with Three-Year kOA Progression

**Figure 2:**
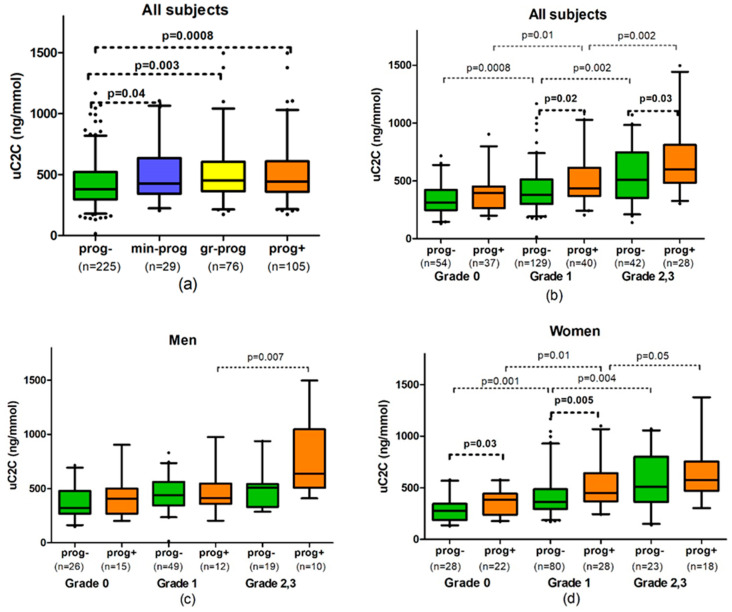
The association of uC2C with radiographic progression of knee osteoarthritis (kOA): (**a**) In all grades in summary; (**b**) in all subjects by global grade of kOA (gOA); (**c**) in men by gOA grades; (**d**) in women by gOA grades. Abbreviations: prog-—non-progressors, subjects without radiographical changes of kOA during 3 years; prog+—progressors, subjects with the progression of kOA; min-prog—minimal progressors, subjects with radiographic worsening of kOA within the same gOA grade; gr-prog—grade progressors, subjects with the progression of gOA ≥ 1 grade; n, numbers. Box-whiskers plot with 5th–95th percentiles, the *p*-value of Mann–Whitney U-test.

We found a significantly higher level of uC2C in progressors compared with non-progressors at T0 (16% difference in the median; *p* = 0.0008; Figure 2a). 

This increase in uC2C level was not only detectable in gr-progressors (19% of difference; *p* = 0.003) but also in minimal progressors (difference of 12%; *p* = 0.04). We did not find a statistically significant difference in uC2C levels between min-progressors and gr-progressors (Figure 2a). 

Similar to the unadjusted data, the uC2C level predicted kOA progression rather well in models adjusted for age, sex, BMI, and gOA grades (OR = 2.34 (1.48–3.68) for uC2C, *p* = 0.0003, AUC = 0.67, Table 3). 

As expected, the uC2C predictive power was even stronger in gr-progressors (OR = 2.8 (1.66–4.72) for uC2C, *p* = 0.0001, AUC = 0.73). Two cofactors in the model contributed to the kOA progression risk: an increase in BMI slightly magnified the risk of the progression of the disease (OR = 1.07 (1.02–1.12); *p* = 0.006), but an increase in gOA grade demonstrated a slight protective effect (OR = 0.67 (0.47–0.96); *p* = 0.03). 

### 3.4. uC2C Prognostic Value for kOA Progressors in Distinct gOA 

We observed that the median uC2C values of progressors were higher than the values of non-progressors at baseline grade 1 of gOA and grade 2/3 of gOA (14% difference; *p* = 0.02 and 18% difference; *p* = 0.03, respectively, Table A1). Analyzing the progression subgroups separately, we found that gr-progressors had a 25–33% higher median uC2C level compared with non-progressors (33% difference in grade 1; *p* = 0.001 and 25% difference in grade 2/3, *p* = 0.03). Moreover, we found no difference in uC2C levels between progressors and non-progressors at the emergence of the disease (baseline grade 0 of kOA). Interestingly, the uC2C levels of emerging kOA were similar to those of non-progressors in grade 1 (*p* = 0.38). There was also no statistically significant difference between the uC2C levels of progressors in grade 1 and non-progressors in grade 2,3 (*p* = 0.72). (Figure 2b).

After adjusting uC2C values for important co-founders of the disease (sex, age, and BMI), we identified the statistically significant predictive power of uC2C in detecting the emergence of kOA (OR = 2.58 (1.08–6.16) for uC2C, without kOA used as comparator group, *p* = 0.03; AUC = 0.64, Table 3). The best model for C2C predictive value was obtained when the long-term control group was compared with progressors (OR = 5.87 (1.71–20.22) for uC2C; *p* = 0.005; AUC = 0.79). Moreover, female sex demonstrated a significant risk for the emergence of kOA (OR = 4.01 (1.12–14.39) compared with the long-term controls; *p* = 0.03), but older age was associated with a protective effect in the model (OR = 0.86 (0.77–0.96) per year of age; *p* = 0.009). 

In more advanced grades, uC2C predicted the OA progression less successfully. If at baseline grade 1, uC2C carried a statistically significant risk of progression (OR = 2.36 (1.19–4.67); *p* = 0.01; AUC = 0.67), then uC2C lacked statistically significant predictive power in patients with baseline grade 2/3 of gOA (Table 3). 

### 3.5. The Sex-Related Differences in Associations with uC2C at Baseline Level and kOA Progression

Several differences in the uC2C prognostic value of kOA between men and women were observed. 

In women, the levels of uC2C were found to be 24–30% higher in progressors compared with non-progressors (*p* = 0.0009 for progressors and *p* = 0.001 for gr-progressors, Table A1). At the starting point, the uC2C median value was up to 72% higher in women with emerging kOA compared with the value of the long-term control group (*p* = 0.009; Figure 2d). However, the difference in uC2C level between progressors and non-progressors decreased in more advanced grades of kOA in women: 24% in gOA grade 1 at baseline (*p* = 0.005) but only 13% in grade 2/3 of gOA (*p* > 0.05).

By contrast, no significant difference in uC2C levels was observed between male progressors and non-progressors (Figure 2c). The exception was the min-progressors’ subgroup of men with baseline grade 2/3 of gOA, which demonstrated significantly higher uC2C in comparison with non-progressors of the same baseline gOA (833.0 (606.0–1019.5) vs. 507.0 (345.0–541.5) ng/mmol, *p* = 0.02, Table A1).

The mean values of uC2C in male non-progressors were significantly higher than in female non-progressors; however, the uC2C levels did not differ in the progressors of both sexes (Table A1).

After adjusting for age, BMI, and gOA stage, uC2C showed statistically significant predictive power in detecting the kOA progression equally in both sexes (OR = 2.59 of uC2C in men and OR = 2.22 in women, Table 3). Moreover, BMI and gOA stage demonstrated significant association with the progression of kOA in women. However, by analyzing the baseline gOA grades separately as subgroups, we did not find a statistically significant predictive power of uC2C in men. In women, uC2C demonstrated extremely strong power to predict emerging kOA if the disease progressed from grade 0 to 1, and progressors were compared with the long-term control group (OR = 23.0 (2.2–245)) of uC2C; the best model with AUC = 0.91, Table 3). In more advanced kOA (grades 2 and 3 of gOA), the predictive power of uC2C was less similar to non-adjusted models.

## 4. Discussion

Our observations revealed that the uC2C levels were positively associated with kOA progression assessed by the Nagaosa–Doherty grading system of radiographs. This result is in line with the previous studies on other populations where the same uC2C detection method was used [8,12,13]. Different from many previously published case-control studies, the majority of the subjects of our population study were middle-aged and had mild kOA radiographic findings. The Nagaosa–Doherty grading system allows better evaluation of osteophytes, and osteophytosis was the main radiographic finding at lower grades as well as the main additional finding among kOA progressors. As mentioned in a systematic review, due to the heterogeneity of both OA-phenotypes and evaluated radiographic signs among the publications, meta-analysis of the studied biochemical markers was not possible [23]. The lack of clear consensus on a definition of radiographic progression of OA or clinical endpoint creates a huge challenge concerning defining and validating the biomarkers. The radiographic outcome is usually defined as an increase in Kellgren and Lawrence (KL) grade compared with baseline, but JSN is also frequently used. However, a prior study demonstrated that JSN is insensitive and unsuitable as a progression marker in clinical studies [24]. The investigations into articular cartilage by MRI or arthroscopy have shown that cartilage damage develops years before radiographic JSN [15,25,26]. Moreover, osteophytosis was found to be an early sign of kOA and strongly associated with cartilage damage detected by MRI [27,28,29]. 

In contrast with previous studies that focused on the radiographic changes in the TF compartment, we added the progression in the PF compartment of the knee. Moreover, we evaluated the two different levels of radiographic changes: within-the-grade and over-the-grade. The results demonstrated that higher uC2C values were predictive even within the same gOA grade, defined as minimal radiographic progression of kOA; as expected, uC2C predicted better over-the-grade progression. As shown in a regression model, the increase in BMI slightly magnified the risk of progression of the disease. This finding seems to be consistent with other research that found that participants with higher BMIs had a higher rate of osteophyte progression [29].

Our study is the first to show that uC2C can predict the early development (emergence) of the disease. This finding was particularly pronounced when the long-term control group (twelve years without any sign of kOA) was considered in the model. The result is all the more remarkable, as the three-year follow-up period is relatively short for evaluating ongoing kOA processes radiographically; moreover, the progression of kOA may be nonlinear with intermittent periods of stabilization [20]. We observed that the predictive value of uC2C was less pronounced in more advanced kOA (grades 2 and 3). Although this result may be explained by the smaller size of the group with advanced kOA, it may be a specific feature for uC2C as a biomarker. 

The validation of biomarkers for emerging kOA on the stage of molecular events supports the finding of disease-modifying drugs, where metabolic perturbations are frequently considered reversible [14]. Previously, another Col2 degradation marker, urinary C-terminal telopeptide of collagen type II (uCTX-II), was investigated as an early biomarker of kOA progression; however, the evidence for that marker is still inconclusive [23,30]. Furthermore, comparative studies demonstrated that uC2C and uCTX-II assays have significant differences: the levels of uCTX-II correlated weakly with uC2C [8,31]. uC2C but not uCTXII was associated with pre-radiographic kOA defined by MRI [32]. 

In general, considering our previous observation, the uC2C values seem to depend on at least two factors: existing gOA grade and the presence or absence of radiographic kOA progression [22]. It is therefore likely that in estimating the risk of kOA progression, the baseline gOA grade level of uC2C must be considered. 

Several sex-related differences were also revealed. We demonstrated that uC2C is a promising prognostic marker for emerging kOA in women but not in men. Moreover, the mean values of uC2C in male non-progressors were significantly higher than in female non-progressors, including the results in the long-term control group. This contrasts with a large FNIH/OARSI biomarker consortium study of reference levels suggesting no effect of sex on uC2C [33]. The reason for this contradiction is unclear, but it might be related to different subject characteristics, especially age, which was significantly younger in our study. Moreover, the higher BMI in progressors compared with non-progressors was characteristic in women in the current study. A recent study in the same cohort demonstrated a significant sex-dependent difference in cytokine production, suggesting the presence of sex-related differences in the pathways of kOA pathogenesis [16]. Further studies with larger sample sizes are needed to clarify the role of sex-related differences in uC2C in OA progression.

The present study has obvious limitations. First, our middle-age population cohort allowed us to focus on the progression of early-grade kOA. The low prevalence of the subjects with advanced kOA cases (grade 2–4), particularly in separate gender groups, decreases the statistical power of the models and prevents drawing definitive conclusions in more advanced kOA. To validate the findings, future work should evaluate uC2C in several directions: using a larger population cohort with a higher prevalence of advanced kOA cases and the observations at intermediate timepoints with different imaging modalities; additionally, a longer observation period than three years would be required. Second, we had a low number of cases with isolated JSN progression; thus, we could only indirectly suggest that the increased level of uC2C reflected the risk of subsequent cartilage damage. Despite this, the association of a high level of uC2C with growing osteophytes is considered to be prognostic of the progression of kOA. Third, the progression of osteoarthritis at other sites was not excluded because the radiographs of other joints were not available. It could cause confounding effects with regards to uC2C levels. Finally, we only examined uC2C as a single biomarker although it integrates a number of possible signs of kOA [22]. A combination of various biomarkers would probably improve the prediction of the disease progression [8]. Investigation of different sets of biomarkers would help to explore certain phenotypes of OA [34,35]. 

This study has several strengths. First, the sample included population-based, intentionally recruited participants at risk of knee OA and thus included sufficient numbers of subjects with early-stage kOA. The inclusion of the long-term controls, those without the development of kOA over 12 years, provides a base of non-progressors, which increased the power of the study to detect significant associations between uC2C and emerging kOA. Second, we used the radiographical scoring system described byNagaosa et al. The system offers a detailed and standardized radiographic evaluation of both the TF and PF compartments, including the exact evaluation of osteophytes. Finally, we used a statistical method, logistic regression, which allowed us to consider generally accepted OA risk factors like sex, age, and BMI [36]. 

As mentioned, many aspects need to be considered in the clinical use of OA biomarkers. They can be summarized with the BIPEDS classification of biomarkers (Burden of disease, Investigational, Prognostic, Efficacy of intervention, Diagnostics, and Safety) [37]. uC2C thus plays a specific role in kOA clinical trials [11]. So far, the diagnostic and prognostic value of uC2C has been demonstrated [13,22]. The present study more specifically indicated its prognostic capacity. Further research should continue in the directions referred to above.

## 5. Conclusions

Higher levels of uC2C predict the progression of kOA during the subsequent three years. uC2C is a sensitive biomarker of the progression: it predicts minimal radiographic changes within the same gOA grade characterized by the addition of osteophytes or progression of joint space narrowing in any compartments of the knee. The predictive value of uC2C seems to be strongest in the early or emerging kOA (grades 1 or 0). A more accurate prediction of the risk of kOA progression was found in women than in men. The best prediction value (>90%) was demonstrated when emerging kOA was compared with the long-term control group without the development of kOA over 12 years. In conclusion, uC2C seems to be an excellent prognostic biomarker of emerging kOA at least in women.

## Figures and Tables

**Table 1:** Distribution of the study cohort.

	Grade 0	Grade 1	Grade 2	Grade 3	Overall
Subjects, n, (% all subjects)	91 (27.6)	169 (51.2)	55 (16.7)	15 (4.5)	330
Progressors, n, (%, subjects in the grade)	37 (41)	40 (24)	23 (42)	5 (33)	105
A. gr-progressors, n, (%, subjects in the grade)	37 (41)	24 (14)	15 (27)	-	76
B. min-progressors, n (%, subjects in the grade)	-	16 (10)	8 (15)	5 (33)	29
Non-progressors, n (%, subjects in the grade)	54 (59)	129 (76)	32 (58)	10 (67)	225
Men, n, (% in the grade)	41 (45)	61 (36)	21 (38)	8 (53)	131
Male progressors, n	15	12	7	3	37
Male gr-progressors, n	15	7	4	-	26
Male min-progressors, n	-	5	3	3	11
Male non-progressors, n	26	49	14	5	94
Women, n, (% in the grade)	50 (55)	108 (64)	34 (62)	7 (47)	199
Female progressors, n	22	28	16	2	68
Female gr-progressors, n	22	17	11	-	50
Female min-progressors, n	-	11	5	2	18
Female non-progressors, n	28	80	18	5	131

gOA grade—global grade of knee osteoarthritis (kOA), summary radiographic grade of kOA in the whole joint; Progressors—the subjects with signs of radiographic kOA progression during 3 years; Non-progressors—subjects without radiographical changes of kOA during 3 years; gr-progressors—grade-progressors, subjects with kOA progression of gOA ≥ 1 grade; min-progressors—minimal progressors, subjects with radiographic worsening of kOA within the same gOA grade; n, numbers.

**Table 2:** Description of the study cohort divided by radiographic global grades of knee osteoarthritis (gOA).

	Grade 0	Grade 1	Grade 2	Grade 3	*p*-Value, Difference between Grades	Overall
Mean age in all subjects, years, ± SD	46.1 ±6.5	47.7 ±6.2	49.5 ±6.8	48.6 ±6.6	0.02 *	47.6 ±6.5
Mean age in progressors, years ± SD	46.4 ±6.2	47.8 ±6.2	50.1 ±6.3	52.2 ±3.4	0.007 *	48.0 ±6.2
Mean age in gr-progressors, years ± SD	46.4 ±6.2	49.5 ±5.5	49.4 ±7.0	-	0.06 *	48.0 ±6.3
Mean age in min-progressors, years ± SD	-	45.3 ±6.3	51.4 ±4.9	52.2 ±3.4	0.008 *	48.1 ±6.3
Mean age in non-progressors, years ± SD	45.8 ±6.7	47.6 ±6.2	49.1 ±7.1	46.8 ±7.2	0.09 *	47.4 ±6.5
*p*-value, age difference between the investigated progression groups	0.7 **	0.3 *	0.8 *	0.07 **		0.8 *
BMI, kg/m^2^ ± SD	26.2 ±5.1	28.0 ±4.8	30.5 ±5.8	29.5 ±6.1	1.5 × 10^−5^ *	28.0 ± 5.3
BMI in progressors, kg/m^2^ ± SD	26.8 ±5.2	29.0 ±4.6	33.0 ±6.2	34.2 ±6.2	4.2 × 10^−6^ *	29.3 ±5.8
BMI in gr-progressors, kg/m^2^ ± SD	26.8 ±5.2	30.8 ±3.8	32.9 ±6.2	-	5.0 × 10^−5^ *	29.2 ±5.6
BMI in min-progressors, kg/m^2^ ± SD	-	26.3 ±4.5	33.2 ±6.7	34.2 ±6.2	0.002 *	29.6 ±6.4
BMI in non-progressors, kg/m^2^ ± SD	25.8 ±5.0	27.7 ±4.8	28.7 ±4.8	27.2 ±4.8	0.6 *	27.4 ±4.9
*p*-value, BMI difference between the investigated progression groups	0.4 **	0.02 *	0.01 *	0.06 **		0.02 *
Mean age in men, years ± SD	46.3 ±6.4	46.6 ±7.1	47.2 ±7.5	47.3 ±6.7	0.6 *	46.6 ± 6.9
Mean age in women years ± SD	45.9 ±6.6	48.2 ±5.5	50.9 ±5.9	50.0 ±6.8	0.0003 *	48.2 ± 6.1
*p*-value, age difference between sexes	0.8 **	0.1 **	0.06 **	0.5 **		0.04 **
BMI in men, kg/m^2^ ± SD	27.5 ±5.6	28.1 ±3.8	28.5 ±4.4	30.1 ±6.9	0.1 *	28.1 ± 4.7
BMI in women, kg/m^2^ ± SD	25.2 ±4.4	28.0 ±5.3	31.7 ±6.2	28.8 ±5.5	1.2 × 10^−6^ *	27.9 ± 5.6
*p*-value, BMI difference between sexes	0.03 **	0.8 **	0.03 **	0.7 **		0.7 **
BMI in female progressors, kg/m^2^ ± SD	26.5 ±4.8	29.2 ±5.2	33.8 ±6.8	31.4 ±7.8	0.0004 *	29.5 ±6.1
BMI in female non-progressors, kg/m^2^ ± SD	24.0 ±3.9	27.5 ±5.2	29.9 ±5.1	27.8 ±5.0	5.0 × 10^−5^ *	27.1 ±5.2
*p*-value, BMI difference between female progression groups	0.06 **	0.2 **	0.08 **	0.6 **		0.008 **
Mean knee VAS score (0–10)	1.0 ±2.0	2.3 ±2.5	3.2 ±2.9	3.6 ±3.3	2.2 × 10^−7^ *	2.2 ±2.6

gOA grade—global grade of knee osteoarthritis (kOA), summary radiographic grade of kOA in the whole joint; Progressors—the subjects with signs of radiographic kOA progression during 3 years; Non-progressors—subjects without radiographical changes of kOA during 3 years; gr-progressors—grade-progressors, subjects with kOA progression of gOA ≥1 grade; min-progressors—minimal progressors, subjects with radiographic worsening of kOA within the same gOA grade; n, numbers; SD, standard deviation; BMI, body mass index; VAS, visual analogue scale; * ANOVA; ** *t*-test.

**Table 3:** Adjusted generalized linear models of uC2C for subject groups with progression in the distinct baseline grades of knee osteoarthritis (gOA), multiple logistic regression analysis.

Models		Independent Variables in the Model	All Subjects		Men		Women	
Baseline gOA	Compared Groups		OR (CI 95%)	AUC (CI 95%)	OR (CI 95%)	AUC (CI 95%)	OR (CI 95%)	AUC (CI 95%)
**All grades**	**Progressor,** n = 105 (M = 37) **Non-progressors,** n = 225 (M = 94)	log2(C2C) Age BMI Sex gOA	2.34 (1.48–3.68) *** 0.99 (0.95–1.03) 1.07 (1.02–1.12) ** 1.54 (0.92–2.59) 0.67 (0.47–0.96) *	0.67 (0.61–0.73)	2.59 (1.18–5.70) * 0.96 (0.90–1.02) 1.05 (0.97–1.15) – 0.78 (0.47–1.31)	0.65 (0.55–0.75)	2.22 (1.26–3.92) ** 1.01 (0.96–1.07) 1.07 (1.01–1.14) * – 0.57 (0.35–0.93) *	0.68 (0.60–0.76)
**All grades**	**gr-progressors,** n = 76 (M = 26) **Non-progressors,** n = 225 (M = 94)	log2(C2C) Age BMI Sex gOA	2.80 (1.66–4.72) *** 0.99 (0.95–1.04) 1.08 (1.02–1.14) ** 1.72 (0.95–3.13) 0.34 (0.21–0.55) ***	0.73 (0.67–0.79)	2.23 (0.93–5.35) 0.96 (0.90–1.03) 1.01 (0.92–1.11) – 0.40 (0.20–0.81)*	0.68 (0.57–0.8)	3.27 (1.66–6.46) *** 1.01 (0.95–1.08) 1.11 (1.04–1.20)** – 0.26 (0.13–0.51)***	0.76 (0.68–0.84)
**Grade 0**	**Emerging kOA,** n = 37 (M = 15) **Without kOA,** n = 54 (M = 26)	log2(C2C) Age BMI Sex	2.58 (1.08–6.16) * 0.98 (0.91–1.05) 1.03 (0.94–1.13) 2.09 (0.81–5.37)	0.64 (0.53–0.76)	2.44 (0.63–9.48) 0.95 (0.85–1.07) 0.96 (0.85–1.09) –	0.58 (0.39–0.77)	2.86 (0.85–9.58) 0.99 (0.89–1.09) 1.11 (0.96–1.29) –	0.72 (0.57–0.87)
**Grade 0**	**Emerging kOA,** n = 37 (M = 15) **Long-term control group,** n = 24 (M = 14)	log2(C2C) Age BMI Sex	5.87 (1.71–20.22) ** 0.86 (0.77–0.96) ** 1.00 (0.88–1.13) 4.01 (1.12–14.39) *	0.79 (0.67–0.91)	5.19 (0.79–34.15) 0.84 (0.71–0.99) * 0.90 (0.76–1.07) –	0.7 (0.50–0.90)	22.95 (2.15–245) ** 0.76 (0.60–0.97) * 1.32 (0.95–1.83) –	**0.91** (0.81–1)
**Grade 1**	**Progressors,** n = 40 (M = 12) **Non-progressors,** n = 129 (M = 49)	log2(C2C) Age BMI Sex	2.36 (1.19–4.67) * 0.98 (0.91–1.04) 1.06 (0.98–1.14) 1.56 (0.70–3.48)	0.67 (0.57–0.76)	1.59 (0.49–5.15) 0.96 (0.87–1.05) 1.05 (0.89–1.25) –	0.60 (0.42–0.78)	2.67 (1.18–6.04) * 0.99 (0.91–1.08) 1.05 (0.97–1.14) –	0.68 (0.56–0.80)
**Grade 2,3**	**Progressors,** n = 28 (M = 10) **Non-progressors, (grade 2,3),** n = 42 (M = 19)	log2(C2C) Age BMI Sex	2.22 (0.89–5.55) 1.02 (0.93–1.13) 1.15 (1.03–1.29) * 1.06 (0.33–3.41)	0.77 (0.66–0.88)	11.41 (0.86–151) 0.91 (0.75–1.11) 1.30 (1.00–1.68) –	0.85 (0.70–1)	1.32 (0.45–3.82) 1.10 (0.95–1.26) 1.12 (0.99–1.27) –	0.70 (0.53–0.86)

gOA grade—global grade of knee osteoarthritis (kOA), summary radiographic grade of kOA in the whole joint; Progressors—the subjects with signs of radiographic kOA progression during 3 years; Non-progressors—subjects without radiographical changes of kOA during 3 years; gr-progressors—grade progressors, subjects with kOA progression of gOA ≥1 grade; Emerging kOA—subjects in initial gOA grade 0 with developing kOA changes during 3 years; Without kOA—subjects with no radiographic kOA changes during the whole study period; Long-term control group—gOA grade 0 during 12 years follow-up, no knee symptoms at baseline; OR, odds ratio of double the rise of uC2C; AUC, area under the curve; CI, confidence interval, BMI, body mass index; * *p* < 0.05; ** *p* < 0.01; *** *p* < 0.001; n, numbers; M, numbers in men.

## Data Availability

The data presented in this study are available on request from the corresponding author. The data are not publicly available due to to privacy and ethical restrictions.

## Appendix A

**Table A1:** The association of uC2C with radiographic progression of knee osteoarthritis (kOA). Radiographical changes are divided into grades according to the grading system by Nagaosa-Doherty (2000).

Baseline gOA Grade	Comparison Group	uC2C in All Subjects ng/mmol Medians (Q1–Q3)	*p*-Value between Groups	uC2C in Men ng/mmol Medians (Q1-Q3)	*p*-Value between Groups	uC2C in Women ng/mmol Medians (Q1–Q3)	*p*-Value between Groups	*p*-Value between Genders in the Group
**All grades**	Progressors (n = 105; M = 37)	442.0 (361.0–604.0)	0.0008 *	426.0 (361.0–614.0)	0.16 *	445.5 (364.5–586.8)	0.0009 *	0.8
	gr-progressors (n = 76; M = 26)	451.0 (365.5–589.2)	0.003 **	418.5 (354.2–534.2)	0.54 **	469.0 (370.8–642.2)	0.001 **	0.5
	min-progressors (n = 29; M = 11)	426.0 (356.0–604.0)	0.04 ***	586.0 (413.5–987.5)	0.06 ***	410.5 (337.2–534.2)	0.16 ***	0.13
	Non-progressors (n = 225; M = 94)	380.0 (296.0–520.0)		414.5 (318.2–539.2)		360.0 (282.0–496.5)		**0.03**
**Grade 0**	Emerging kOA (n = 37; M = 15)	395.0 (269.0–449.0)	0.08 *	407.0 (289.0–477.5)	0.57 *	387.0 (253.2–439.5)	0.03 *	0.6
	Without kOA (n = 54; M = 26)	310.5 (249.8–414.8)		322.0 (277.2–474.0)		279.5 (200.8–338.2)		**0.03**
	**Long term control group (n = 24; M = 14)**	**290.5** **(204.2–406.2)**	**0.09 ******	**360.5** **(261.0–468.5)**	**0.65 ******	**225.0** **(176.8–296.0)**	**0.009 ******	**0.02**
**Grade 1**	Progressors (n = 40; M = 12)	434.5 (374.0–607.0)	0.02 *	413.5 (374.5–517.0)	0.83 *	449.0 (374.0–624.8)	0.005 *	0.6
	gr-progressors (n = 24; M = 7)	504.5 (400.0–660.2)	0.001 **	484.0 (391.0–532.0)	0.45 **	527.0 (430.0–688.0)	0.0007 **	0.4
	min-progressors (n = 16; M = 5)	394.5 (304.5–443.5)	0.95 ***	409.0 (282.0–418.0)	0.59 ***	381.0 (321.5–449.0)	0.57 ***	1.0
	Non-progressors (n = 129; M = 49)	380.0 (302–512)		440.0 (346.0–562.0)		362.0 (296.8–473.2)		**0.04**
**Grade 2,3**	Progressors (n = 28: M = 10)	600.0 (504.0–808.2)	0.03 *	640.0 (547.8–1019.5)	0.11 *	576.5 (484.0–732.0)	0.59 *	0.3
	gr-progressors (n = 15; M = 4)	653.0 (547.5–773.0)	0.03 **	574.5 (504.0–834.8)	0.22 **	686.0 (570.5–773.0)	0.18 **	0.6
	min-progressors (n = 13; M = 6)	572.0 (426.0–980.0)	0.21 ***	833.0 (606.0–1019.5)	0.02 ***	454.0 (384.5–565.5)	0.67 ***	**0.03**
	Non-progressors (n = 42; M = 19)	509.0 (360.0–743.5)		507.0 (345.0–541.5)		511.0 (371.5–776.0)		0.5

gOA grade—grade of knee osteoarthritis (kOA), summary radiographic grade of kOA in whole joint; Progressors—the subjects with signs of radiographic kOA progression during 3 years; Non-progressors—subjects without radiographical changes of kOA during 3 years; gr-progressors—global grade progressors, subjects with kOA progression of gOA ≥1 grade; min-progressors—minimal progressors, subjects with radiographic worsening of kOA within the same gOA grade; Emerging kOA—subjects in initial gOA grade 0 with developing kOA changes during 3 years; Without kOA—subjects with no radiographic kOA changes during the whole study period; Long-term control group—gOA grade 0 during 12 years follow-up, no knee symptoms at baseline; Q1, first quartile, Q3, third quartile; P-value of Mann–Whitney U-test; Comparisons of uC2C values in different groups: * Progressors vs. non-progressors (emerging kOA vs without kOA in gOA grade 0); ** gr-progressors vs. non-progressors; *** min-progressors vs. non-progressors; **** Emerging kOA vs. control group; # men vs. women.
